# Molecular Identification of *Borrelia afzelii* from Ticks Parasitizing Domestic and Wild Animals in South Korea

**DOI:** 10.3390/microorganisms8050649

**Published:** 2020-04-29

**Authors:** Min-Goo Seo, Oh-Deog Kwon, Dongmi Kwak

**Affiliations:** 1 Veterinary Drugs and Biologics Division, Animal and Plant Quarantine Agency, 177 Hyeoksin 8-ro, Gimcheon, Gyeongbuk 39660, Korea; koreasmg@korea.kr; 2 College of Veterinary Medicine, Kyungpook National University, 80 Daehak-ro, Buk-gu, Daegu 41566, Korea; odkwon@knu.ac.kr; 3 Cardiovascular Research Institute, Kyungpook National University, 680 Gukchaebosang-ro, Jung-gu, Daegu 41944, Korea

**Keywords:** *Borrelia afzelii*, phylogeny, tick, domestic and wild animals

## Abstract

Lyme borreliosis is one of the most prevalent tick-borne infectious zoonotic diseases caused by spirochetes of the *Borrelia burgdorferi* sensu lato group. The present study assessed the risk factors and prevalence of Lyme borreliosis in ticks parasitizing domestic and wild animals. A total of 589 ticks (329 tick pools) collected from animals were identified as *Haemaphysalis longicornis*, (85.7%), *H. flava* (10.0%), and *Ixodes nipponensis* (4.3%) using morphological and molecular methods in South Korea. In this study, the 5S–23S gene sequences of *B. afzelii* (6/329, 1.8%) were detected in ticks taken from mammals, including ticks from horses (2/147 pools, 1.4%), wild boar (1/19 pools, 5.3%), native Korean goats (NKG, 2/34 pools, 5.9%), and Korean water deer (1/129 pools, 0.8%). Unfortunately, *ospA*, *pyrG*, and *flagellin* genes were not able to be amplified in the present study. To our knowledge, our results are the first inclusive data available for *B. afzelii* circulation in several tick species taken from NKG, horses, and wild boar in South Korea. We believe that the current findings extend our knowledge of the distribution and possible vector spectrum of *Borrelia* spp. We recommend continuous evaluation of the potential public health threat posed by *Borrelia* infected ticks.

## 1. Introduction

Numerous emergent tick-borne pathogens (TBPs) had been circulating in ticks and animals long before their identification as causes of clinical diseases [1]. The global hazard of TBPs is increasing and raising public health concerns, as novel pathogens have been continuously detected during the last two decades [2]. There have been changes in populations of wild animals, as well as the ticks and pathogens that they transmit; therefore, the risk of TBPs is increasing globally [3].

Lyme borreliosis is one of the most prevalent tick-borne infectious zoonotic diseases caused by spirochetes of the *Borrelia burgdorferi* sensu lato group. It is transmitted through the bite of infected *Ixodes* ticks, and mainly occurs in North America, Europe, and Asia. *B. burgdorferi* s.l. group includes at least 19 species [4]. Of these, three species (*B. burgdorferi* sensu stricto, *Borrelia bissettii*, and *Borrelia andersonii*) have been recognized in North America, five species (*B. burgdorferi* s.s., *Borrelia valaisiana*, *Borrelia garinii*, *Borrelia lusitaniae*, and *Borrelia afzelii*) have been identified in Europe, and seven species (*Borrelia japonica*, *B. garinii*, *B. valaisiana*, *Borrelia tanukii*, *B. afzelii*, *Borrelia turdi*, and *Borrelia sinica*) have been recognized in Asia. Differentiation and identification of *B. burgdorferi* s.l. species can be achieved by molecular methods [5]. At least three *B. burgdorferi* s.l. species, i.e., *B. burgdorferi* s.s., *B. afzelii*, and *B. garinii* are the main etiological agents of human Lyme disease [5]. Among the other species, *Borrelia hermsii* is the main cause of tick-borne relapsing fever in western North America [6]. *Borrelia spielmanii* has been identified in early skin disease, *B. bissettii* and *B. valaisiana* have been identified in specimens from single cases of Lyme borreliosis, and the clinical role of *B. lusitaniae* remains to be substantiated [7]. Other species are not pathogenic to humans, such as the America-specific *B. andersonii* [8], the Japan-specific *B. japonica* [9], *B. tanukii* and *B. turdi* [10], and the China-specific *B. sinica* [11].

Due to recent rises in outdoor activities (e.g., hiking, farming, and military acts) and as a consequence of environmental changes, such as global warming, humans and animals are more likely to be exposed to ticks [3]. Considering the ecology of local tick species that parasitize domestic and wild animals and recognizing the potential pathogens (e.g., Lyme borreliosis) that they carry are of paramount significance in public health. Previous studies have examined the presence of Lyme borreliosis in South Korea by studying ticks, humans, and rodents via molecular methods [12,13,14,15,16].

This study measured the risk factors of Lyme borreliosis, and its prevalence among ticks taken from Korean water deer (KWD, *Hydropotes inermis argyropus*), native Korean goats (NKGs, *Capra hircus coreanae*), horses, and wild boar. In addition, to further clarify the nature and significance of tick infestations in domestic and wild animals in South Korea, we measured the current prevalence of tick infestations and the chief tick species responsible for those infestations.

## 2. Materials and Methods

### 2.1. Ethics

The present study was conducted between 2015 and 2019 and did not receive approval from the Institutional Animal Care and Use Committee at Kyungpook National University because this committee evaluates laboratory animals maintained in indoor facilities and does not regulate research with outdoor animals. Since the law of “Act on the Prevention of Contagious Animal Disease (Amendment Act 2015)” operated in South Korea, national and local veterinary institutes performed control measures in accordance with annual infectious animal disease control programs. Collection of ticks from NKG and horses were performed in accordance with this project by practicing veterinarians at local, government-run veterinary institutes during monitoring, surveillance, treatment, or regular check-ups after the receipt of oral consent from the farm owner. Removal of ticks did not hurt or cause any physical injury to animals.

Tick collection from wild animals was performed as follows: ticks were collected from KWD that had been killed by vehicles on highways or roadsides. This process was supervised by the National Institute of Environmental Research, Ministry of Environment, South Korea. To satisfy the OIE requirements for the surveillance of feral pigs and wild boars in classical swine fever-free countries, wild boars were hunted or captured in cooperation with the Korean Pork Producers Association and the National Veterinary Institutes of South Korea since 2010 [17]. Wild boars were hunted or captured following guidelines for the capture of hazardous wild animals as published by the Korean Ministry of Environment with the help of the Korean Wildlife Management Association. Ticks were collected from the dead bodies of wild boars.

### 2.2. Tick Collection and Species Identification

A total of 589 ticks were collected from domestic and wild animals in four regions across the country: northern (Gyeonggi (20) and Gangwon (45)), central (Chungbuk (26), Chungnam (23), and Gyeongbuk (59)), southern (Jeonbuk (74), Jeonnam (78), and Gyeongnam (49)), and Jeju Island (215), between May and September of 2015–2019. The documented number of horses reared in South Korea was 27,243 in 2018; 57.5% of these animals were reared on Jeju Island [18]. Thus, ticks from horses were harvested from Jeju Island. The total number of NKGs reared in South Korea was reported to be 542,744 from 14,644 farms in 2018; 50.4% of these animals were reared in southern areas [19]. Therefore, most ticks from NKGs were collected from southern areas. On the other hand, ticks from wild animals (KWD and wild boars) were harvested throughout the country.

For domestic animals (horses and NKGs), first, 36 farms were randomly selected from 97 farms using a simple random sampling method during monitoring, surveillance, and treatment. Second, within each selected farm, we randomly selected 2–3 domestic animals per farm from 36 farms by a simple random sampling method.

One to eleven ticks per animal were collected from animals, including 71 horses, 10 wild boars, 28 NKGs, and 42 KWD. Ticks were collected from animals by their body and mouth parts using fine forceps and were gently removed. The ticks were then stored in 70% ethanol until further use. The collected ticks were initially identified according to their morphological characteristics [20], with further classification done according to the molecular methods described below to avoid potential mistakes in morphologic identification. Afterward, the ticks were pooled by species and developmental stage (nymph versus adult) into 329 tick pools, with one to four ticks per pool.

### 2.3. Molecular Detection of Ticks and TBPs

Genomic DNA was extracted from the ticks using a commercial DNeasy Blood and Tissue Kit (Qiagen, Melbourne, Australia), according to the manufacturer’s instructions. The AccuPower Profi PCR Premix Kit (Bioneer, Daejeon, South Korea) was used for PCR amplification. Molecular identification of tick species was conducted by amplifying sequences of the mitochondrial cytochrome c oxidase subunit I (*COI*) gene using specific primers [21].

A nested PCR was performed with primers designed to amplify the variable spacer region between two conserved structures, 5S–23S rRNA of *Borrelia* [15,22], expected to yield 226–266 bp fragments depending on the species.

Additionally, for molecular characterization, positive samples were submitted to amplify the outer surface protein A (*ospA*), CTP synthase (*pyrG*), and *flagellin* genes, as previously described [15,16].

### 2.4. DNA Cloning, Nucleotide Sequencing, and Phylogenetic Analysis

For positive PCR products, DNA cloning was done using pGEM-T Easy vectors (Promega, Madison, WI, USA) and *Escherichia coli* DH5α-competent cells (Thermo Fisher Scientific, Wilmington, DE, USA). Two bacterial colonies from each PCR product were followed by nucleotide sequencing using the multiple sequence alignment program CLUSTAL Omega (v. 1.2.1), and the alignment was edited using BioEdit (v. 7.2.5). Phylogenetic analysis was performed using MEGA (v. 6.0), and was based on the maximum likelihood method with the Kimura two-parameter distance model. The aligned sequences were analyzed using a similarity matrix. The stability of the trees was estimated via bootstrap analysis using 1000 replicates.

## 3. Results

### 3.1. Identification of Ticks

A total of 589 ticks (329 tick pools) involving two genera and three species (*Haemaphysalis longicornis*, *Haemaphysalis flava*, and *Ixodes nipponensis*) were collected from mammals, including 215 ticks (147 tick pools) from horses, 34 ticks (19 tick pools) from wild boars, 97 ticks (34 tick pools) from NKGs, and 243 ticks (129 tick pools) from KWD. Among the 329 tick pools, 55 nymph and 274 adult pools were made. No larval ticks were detected in this study.

*cH. longicornis* was distributed throughout the country including mainland Korea and Jeju Island, whereas *H. flava* and *I. nipponensis* were distributed in the central and southern areas. Among these, the ticks positive for *B. afzelii* were collected from different areas, including one *H. longicornis* from NKG in the southern area, two *H. longicornis* from horses in Jeju Island, one *H. flava* from a wild boar in the southern area, one *I. nipponensis* from NKG in the southern area, and one *I. nipponensis* from KWD in the central area.

Universal primers for the *COI* gene were used to amplify 710 bp fragments in the tick samples. The *COI* gene in representative ticks was sequenced and analyzed to eliminate potential mistakes in morphological identification, especially in nymphs. The *COI* gene sequences obtained in this study were classified into three groups according to nucleotide identity. Morphological and molecular characteristics were congruent with each other in species identification. In addition, the sequences of two bacterial colonies from each PCR product were 100% identical each other. The three species within the 329 tick pools shared close genetic relationships with *H. longicornis* (98.2–100% nucleotide identity), *H. flava* (99.7–100% nucleotide identity), and *I. nipponensis* (99.2–100% nucleotide identity).

**Figure 1:**
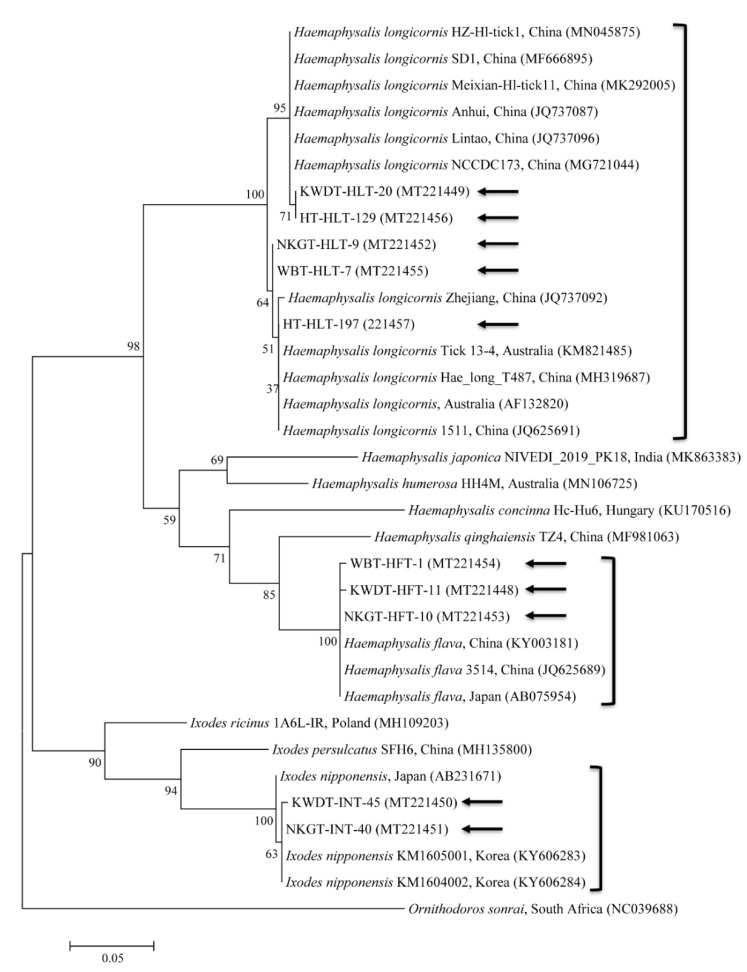
Molecular identification of ticks, collaborated with morphologic identification, according to phylogenetic analysis using the maximum likelihood method with the mitochondrial cytochrome c oxidase subunit I gene. *Ornithodoros sonrai* was used as the outgroup. Black arrows indicate the sequences analyzed in this study. GenBank accession numbers for other sequences are shown with the sequence name. Branch numbers indicate bootstrap support (1000 replicates). The scale bar indicates the number of substitutions per nucleotide.

Among the sequences of the 329 tick pools, 10 representative sequences were selected from different animal ticks (three from KWD ticks, three from NKG ticks, two from wild boar ticks, two from horse ticks) for phylogenetic analysis. A phylogenetic tree was created according to the *COI* genes of several ticks deposited in GenBank, and the ticks collected in this study were classified into three clades related to the following three species (Figure 1): *H. longicornis* (85.7%, 282/329 pools), *H. flava* (10.0%, 33/329 pools), and *I. nipponensis* (4.3%, 14/329 pools) (Table 1).

The 10 representative tick sequences found in this study were submitted to GenBank with the following accession numbers: MT221449, MT221452, MT221455–MT221457 (*H. longicornis*), MT221448, MT221453, MT221454 (*H. flava*), and MT221450–MT221451 (*I. nipponensis*).

### 3.2. Identification of Borrelia spp.

In this study, the 5S–23S gene sequences of *B. afzelii* (6/329, 1.8%) were detected in ticks taken from mammals (Table 1), including ticks from horses (2/147 pools, 1.4%), wild boars (1/19 pools, 5.3%), NKGs (2/34 pools, 5.9%), and KWD (1/129 pools, 0.8%). Unfortunately, *ospA*, *pyrG*, and *flagellin* genes were not able to be amplified in the present study.

With regard to each tick from animals, *B. afzelii* was detected for 3 (0.9%) *H. longicornis* adults, 1 (3.6%) *H. flava* adult, and 2 (20.0%) *I. nipponensis* adults; the results included 2 (1.4%) *H. longicornis* adults from horses, 1 (7.1%) *H. flava* adult from wild boar, 1 (10.0%) *H. longicornis* adult and 1 (50.0%) *I. nipponensis* adult from NKGs, and 1 (12.5%) *I. nipponensis* adult from KWD.

### 3.3. Molecular and Phylogenetic Analyses

**Figure 2:**
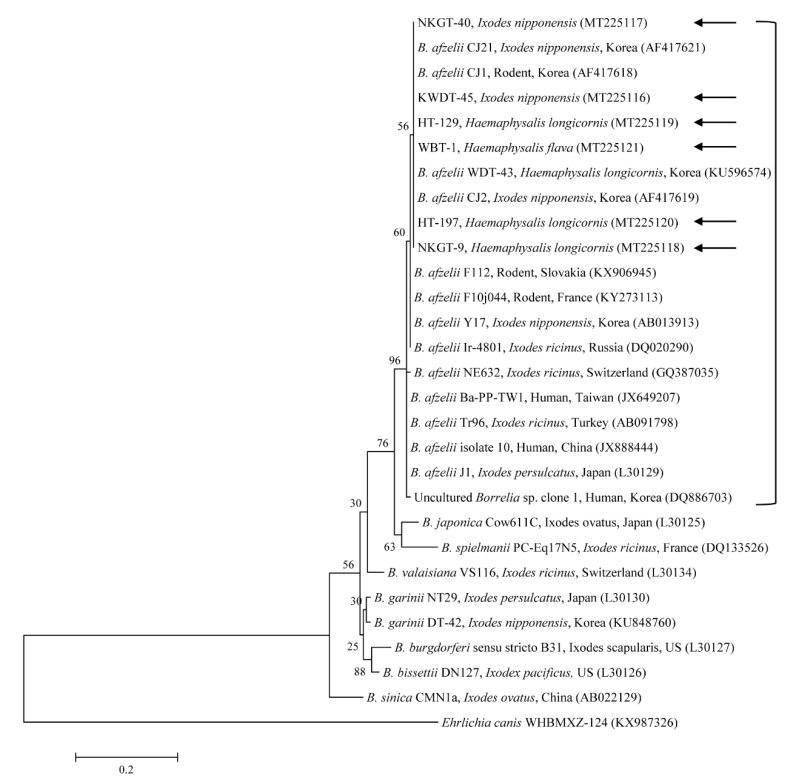
Phylogenetic tree constructed using the maximum likelihood method and based on the 5S–23S rRNA nucleotide sequences of *Borrelia* spp. *Ehrlichia canis* was used as the outgroup. Black arrows indicate the sequences analyzed in this study. *B. afzelii*, *B. bissettii*, *B. burgdorferi* s.s., *B. garinii*, *B. spielmanii*, and *B. valaisiana* infect humans with pathogenic potential, causing Lyme borreliosis. *B. japonica* and *B. sinica* have not yet been reported in or isolated from humans. GenBank accession numbers for other sequences are shown with the sequence name. Branch numbers indicate bootstrap support (1000 replicates). The scale bar indicates the number of substitutions per nucleotide.

Phylogenetic analyses showed that the 5S–23S (Figure 2) nucleotide sequences of *Borrelia* spp. were clustered with previously documented sequences.

The six sequences of *B. afzelii* found in the present study shared 99.6–100% identity with the 5S–23S gene sequence. They also shared 98.8–100% identity with the 5S-23S gene sequences in previously reported *B. afzelii* isolates. The six sequences of *B. afzelii* found in this study were submitted to GenBank. Since the sequences of two bacterial colonies from each PCR product were 100% identical each other, only one for each sample was submitted to GenBank with the accession numbers MT225116–MT225121.

## 4. Discussion

In the present study, *H. longicornis* (85.7%), *H. flava* (10.0%), and *I. nipponensis* (4.3%) were found in domestic and wild animals. *H. longicornis* was the most common species. These findings are consistent with the results of previous South Korean studies. Kim et al. (2014) [3] found 106 *H. longicornis* (one adult female, four nymphs, and 101 larvae), five *H. flava* (four adult females and one nymph), and four *I. nipponensis* (one adult male and three adult females) on water deer. Chae et al. (2019) [23] found 569 *H. longicornis* (369 adult males, 162 adult females, and 38 nymphs) on goats as well as 297 *H. longicornis* (44 adult males, 185 adult females, and 68 nymphs), 118 *H. flava* (94 adult males and 24 adult females), one *I. nipponensis* (one adult male), and 49 *Amblyomma testudinarium* (30 adult males and 19 adult females) on wild boars. Seo et al. (2016) [24] found 231 *H. longicornis* adults on horses. According to a previous study in South Korea, *H. longicornis* was more frequently collected in April–August, while *H. flava* and *I. nipponensis* were collected more frequently in April–July and October [25]. The seasonal prevalence of TBP infections correlates with tick populations and activity in the summer and autumn periods and is of key importance in the transmission of TBPs [26]. For instance, ticks transmitting Lyme disease are distributed throughout South Korea and are primarily seen from May to November [26]. In the present study, all ticks were collected from May to September, and all six positive ticks of *B. afzelii* were collected in the summer. Among these ticks, *B. afzelii* was detected in different areas, including 2.1% (1/48) in the central area, 3.1% (3/98) in the southern area, and 1.4% (2/147) from Jeju Island. In the present study, although ticks were collected in limited numbers and from limited regions, the distribution and abundance patterns of ticks showed similar patterns with those of a previous study [26], showing that ticks and TBPs are more frequent in the summer and southern area. These findings indicate that additional seasonal and geographical studies are needed to fully understand tick populations and to clarify the distribution of TBPs in mammals.

*COI* sequences from collected *H. longicornis* shared 98.2–99.6% nucleotide identity with a known *COI* sequences of *H. longicornis* (Figure 1). The *COI* sequences from collected *H. flava* and *I. nipponensis* shared 97.2–98.7% and 97.9–98.5% nucleotide identity with known *COI* sequences, respectively (Figure 1). *H. longicornis* is naturally collected from grasses and herbaceous vegetation, *H. flava* is naturally collected from forests, and *I. nipponensis* is collected from both habitats in South Korea [25]. All three ticks identified in this study are vectors and hosts for many TBPs and can transmit them to humans and animals in South Korea [13,14,27,28].

In South Korea, *B. burgdorferi* s.l. was first identified as both *B. afzelii* and *B. garinii* collected from *I. persulcatus* and wild rodents in 1992 [29], and the first human case of Lyme disease was also reported in 1993 [30]. *B. afzelii* has also been isolated from *I. nipponensis*, *I. granulatus*, and wild rodents [31]. Until now, many *Borrelia* spp. were detected: *B. afzelii* was first detected molecularly in the sera of patients with febrile clinical diseases (2.8%, 8/283) [12], *B. burgdorferi* s.l. was detected in *I. nipponensis* ticks (3.8%, 16/420 pools) collected from grass and on rodents [13], and *B. afzelii* was detected in *I. nipponensis* ticks (0.6%, 3/484) collected from grass [16]. Despite shortcomings of individual studies on the vector competence of different genera of *Dermacentor*, *Amblyomma*, and *Haemaphysalis* ticks for *B. burgdorferi* s.l. spirochetes, the collective evidence shows that ticks of these genera are unlikely to contribute more than minimally, if at all, to transmission of Lyme borreliosis spirochetes [32]. However, this needs a big sample size for a statistically robust evaluation. For example, lack of *B. burgdorferi* s.s. infection in 520 examined *H. longicornis* nymphs fed as larvae on infectious hosts still resulted in an upper 95% confidence limit estimate for infection prevalence of 0.7% [33]. Therefore, the effort needed to conclude that a given tick species is improbable to be a vector is considerable. An additional study is required to fully understand the potential of *Haemaphysalis* tick as a vector of *B. burgdorferi* s.l. spirochetes. Officially in South Korea, Lyme disease was designated a group 3 infectious disease. Surveillance has been conducted by Korea Centers and Disease Control and prevention has been overseen according to the Infectious Disease Control and Prevention Act since 2010. Subsequently, the cases of this disease have a gradually increasing tendency, and recently 9, 27, 31, 23, and 23 cases were reported each year from 2015 to 2019, respectively [34].

The present study detected the presence of *B. afzelii* in three different tick species that infest domestic and wild animals in South Korea. Even if KWD, horses, wild boars, and NKGs are not main reservoirs of *B. afzelii*, they may be sentinels for the distribution and increase in prevalence of *Borrelia* spirochetes, and they can serve as reservoirs for infection for other domestic and wild animals. As for the detection of this pathogen in an individual, the presence of a blood-feeding tick on an individual by no means proves or even proposes its role as a vector and a series of special demands would need to be studied to prove vector suitability [16]. Normally, *B. burgdorferi* s.l. is transmitted by *Ixodes* spp. [35]. Further investigation needs to be conducted to determine whether *H. longicornis* and *H. flava* ticks are competent vectors of *B. afzelii* and whether KWD, horses, wild boars, and NKGs are competent reservoirs of *B. afzelii*. 

KWD are one of the greatest generally distributed wild ungulates in South Korea and are known to be a critical natural reservoir hosts of numerous TBPs that influence wildlife, humans, and domestic animal populations [27]. Owing to the closeness of KWD to domestic animals, they can transmit TBPs to these animals. In South Korea, *B. afzelii* was previously identified in *H. longicornis* tick (2.1%, 1/48 pools) taken from KWD [15]. In the present study, one *I. nipponensis* adult tested positive for *B. afzelii.* To our knowledge, this is the first study to report the presence of *B. afzelii* in *I. nipponensis* ticks taken from KWD in South Korea.

The NKG is a distinct breed of goat mainly raised for natural health supplements and meat in South Korea, and it is known to be an important natural reservoir host of some TBPs [28]. As NKGs are reared near domestic animals, they can transmit TBPs to these animals. Moreover, TBP infections in goats may be ignored because of their low economic value in the NKG production industry in South Korea [28]. In North Korea, *B. theileri* was identified in *H. longicornis* ticks (1.0%, 3/292) taken from goats [14]. In the present study, one *H. longicornis* adult and one *I. nipponensis* adult tested positive for *B. afzelii.* To our knowledge, this is the first study to report the presence of *B. afzelii* in ticks taken from NKG in South Korea.

Ticks are responsible for a broad range of health and welfare problems in horses. Tick-borne equine piroplasm infections in horses limit worldwide trade and sporting activities concerning these animals [36]. Horses are mainly used for racing activities and regularly kept for leisure; so, humans are highly at risk of exposure to tick bites that previously fed on TBP infected horses. Workers in the horse industry are a high-risk group of TBP infections, including horse keepers, jockeys, trainers, and veterinarians. *B. burgdorferi* infection is common in horses living in Lyme endemic regions and the geographic range for exposure is growing, and naturally occurring syndromes attributed to *B. burgdorferi* infection in horses include uveitis, neuroborreliosis, and cutaneous pseudolymphoma [37]. Controversies surrounding Lyme disease and *B. burgdorferi* infection exist in both humans and horses, and this is particularly true for those patients with relapsing or chronic nervous system signs, muscle or joint pain and lethargy that remain seropositive for *B. burgdorferi* after antibiotic treatment [37]. The high number of confirmed cases of Lyme diseases in humans and the strong evidence for geographic expansion of *B. burgdorferi* infection in both horses and humans [37]. Among *B. burgdorferi* s.l. species, human Lyme disease can be caused by *B. afzelii* [5], and *B. afzelii* was detected molecularly in the sera of patients with febrile clinical diseases in South Korea [12]. However, *B. afzelii* infection has not yet been reported in horses. In other countries, *B. burgdorferi* has been reported in horses (2.3%, 7/300) from Italy [38]. In South Korea, the horse industry continues to grow each year [18]. Thus, it is important to reduce or prevent the spread of zoonotic diseases between humans and horses. In South Korea, *B. burgdorferi* was only detected in horses (5.2%, 38/727) via enzyme-linked immunosorbent assay (ELISA) test [39]. In the present study, two *H. longicornis* adults tested positive for *B. afzelii*. To our knowledge, this is the first study to report the presence of *B. afzelii* in ticks taken from horses in South Korea.

Wild boars are endemic to many countries in the world. Recreational hunting of wild boar and eating of wild boar meat in some regions of the world further provides sufficient opportunities for direct human contact with wild boar, and thus has created an ideal environment for transmission of many pathogens affecting wild boar that could possibly be introduced to domestic animals and humans [40]. In South Korea, wild boars are regularly encountered and observed in urban regions, and the trapping of these animals grew each year. The identification of *B. afzelii* in wild boar is a significant epidemiological result [41], and this hypothetical association between wild boar and *B. afzelii* has previously been suggested, since the DNA of both has been detected concurrently in the blood meal of ticks [42]. *Borrelia* spp. have been reported in wild boars and related ticks from other countries, including *Borrelia* sp. in *Ixodes ricinus* ticks (8.3%, 4/48) on wild boars in Netherlands [43], *B. afzelii* on wild boars (3.3%, 3/90) in Portugal [41], and *Borrelia* sp. on wild boars (8.4%, 16/190) and *Borrelia* sp. in larval tick (3.1%, 1/32 pools) from wild boars and sika deer in Japan [44]. However, *Borrelia* spp. has not yet been reported in wild boar and related ticks in South Korea. In the present study, one *H. flava* adult tested positive for *B. afzelii.* To our knowledge, this is the first study to report the presence of *B. afzelii* in ticks taken from wild boar in South Korea.

The identification of *Borrelia* genospecies is important because the two major clades of *Borrelia* spirochetes, the causative agents of relapsing fever *Borrelia* and Lyme borreliosis *B. burgdorferi* s.l., are morphologically indistinguishable and are hard to differentiate biochemically [45]. Identification is important because the symptoms of Lyme borreliosis and pathogenicity of the *Borrelia* genospecies in humans differs, with each genospecies being related to a specific illness, and different clinical symptoms may occur in different geographic regions [12]. Normally, patients with Lyme arthritis are more frequently infected with *B. burgdorferi* s.s., those with acrodermatitis chronica atrophicans with *B. afzelii*, and those with neuroborreliosis with *B. garinii* [12]. *B. burgdorferi* s.s., *B. afzelii*, and *B. garinii* are pathogenic to humans in Europe, whereas *B. burgdorferi* s.s. is recognized to cause human infection in North America [5]. Thus, arthritis appears to be more common in North American patients, whereas acrodermatitis chronica atrophicans, lymphocytoma, and encephalomyelitis have been mainly observed in Europe [5].

The results of present study, which show that *B. afzelii* was the prevalent genospecies in ticks taken from domestic and wild animals in the examined regions, are consistent with the previous results that show the prevalence of *B. afzelii* in ticks, humans, and wild rodents collected in various regions of South Korea. 

The 5S–23S, *flagellin*, *ospA*, and *ospC* genes have all been used to classify the genospecies and genetic variety of *Borrelia* spirochetes [4]. The 5S-23S gene is extremely conserved and specific to each genospecies [22]. Although, several other genes were not identified in the present study, identified sequences of the 5S–23S gene indicated that the detected *Borrelia* spirochetes had a high genetic identity compared with *B. afzelii*, and that the sequences were obviously different from the 5S–23S sequences of clades of other *B. burgdorferi* s.l. species. 

Limited data are available about the *Borrelia* spp. of ticks that exist on wild and domestic animals in South Korea. Although limited numbers of ticks were collected from wild and domestic animals in this study, tick distribution and abundance patterns were similar to those in previous studies [3,23,24], showing the predominance of *H. longicornis*, followed by *H. flava* and *I. nipponensis*. Therefore, we believe that the current findings extend our knowledge of the distribution and possible vector spectrum of *Borrelia* spp.

## 5. Conclusions

The climate of South Korea is gradually shifting to a subtropical one due to global warming. The emergence of endemic TBPs might be associated with climate-driven changes to their geographic ecology and range. To our knowledge, our study includes the first comprehensive dataset available regarding *B. afzelii* circulation in several ticks parasitizing wild and domestic animals in South Korea and can be considered a pilot study to assess possible risk for humans. Removing ticks from animals is critical because these species carry further risks to both animals and human public health. The present findings extend our knowledge of the distribution and probable vector spectrum of *Borrelia* spp. and suggest that ticks are a potential reservoir for *Borrelia* spp. transmission to other animals and humans via bites. Study of tick infestation risk is significant, especially from zoonotic and public health perspectives.

## Figures and Tables

**Table 1:** Prevalence of *Borrelia afzelii* detected by PCR in ticks collected from several mammals in South Korea, 2015–2019.

Species	Region	Stage	No. Tick Positive/Tick Pool (%)				
			Horse	Wild Boar	Native Korean Goat	Korean Water Deer	Total
***Haemaphysalis longicornis***	Northern	Nymph	0	0	0	0/10	0/10
		Adult	0	0/2	0	0/24	0/26
	Central	Nymph	0	0	0	0/11	0/11
		Adult	0	0/1	0	0/22	0/23
	Southern	Nymph	0	0	0/12	0/13	0/25
		Adult	0	0/2	1/10 (10)	0/28	1/40 (2.5)
	Jeju Island	Nymph	0	0	0	0	0
		Adult	2/147 (1.4)	0	0	0	2/147 (1.4)
	Subtotal	Nymph	0	0	0/12	0/34	0/46
		Adult	2/147 (1.4)	0/5	1/10 (10)	0/74	3/236 (0.9)
*Haemaphysalis flava*	Central	Nymph	0	0	0	0/1	0/1
		Adult	0	0/4	0	0/4	0/8
	Southern	Nymph	0	0	0/3	0/1	0/4
		Adult	0	1/10 (10)	0/4	0/6	1/20 (5)
	Subtotal	Nymph	0	0	0/3	0/2	0/5
		Adult	0	1/14 (7.1)	0/4	0/10	1/28 (3.6)
*Ixodes nipponensis*	Central	Nymph	0	0	0	0/1	0/1
		Adult	0	0	0	1/4 (25)	1/4 (25)
	Southern	Nymph	0	0	0/3	0	0/3
		Adult	0	0	1/2 (50)	0/4	1/6 (16.7)
	Subtotal	Nymph	0	0	0/3	0/1	0/4
		Adult	0	0	1/2 (50)	1/8 (12.5)	2/10 (20)
	Total		2/147 (1.4)	1/19 (5.3)	2/34 (5.9)	1/129 (0.8)	6/329 (1.8)
